# Effect of enhanced masticatory force on OPG, RANKL and MGF in alveolar bone of ovariectomized rats

**DOI:** 10.1590/1678-7757-2019-0409

**Published:** 2020-04-03

**Authors:** Zongmin Ma, Shuxian Li, Yuchen Sun

**Affiliations:** 1 Dalian University Mechanical Engineering College Dalian China Dalian University, Mechanical Engineering College, Dalian, China.; 2 Dalian University Graduate School Dalian China Dalian University, Graduate School, Dalian, China.

**Keywords:** Masticatory force, Alveolar bone loss, Osteoprotegerin, Receptor activator of nuclear factor kappa B ligand, Mechano–growth factor, rat

## Abstract

Menopause induces oral bone loss, leading to various oral diseases. Mastication importantly affects bone metabolism in the jawbone.

**Objective:**

To analyze the effect of enhanced masticatory force on osteoprotegerin (OPG), receptor activator of nuclear factor kappa B ligand (RANKL), and mechano–growth factor (MGF) in alveolar bone of ovariectomized rats and to study the mechanics mechanism of the alveolar bone of ovariectomized rats response to enhanced masticatory force.

**Methodology:**

Thirty Sprague Dawley rats were randomly divided into three groups: sham–operation group (fat around the removed ovary + normal hard diet), model group (ovariectomy + normal hard diet), and experimental group (ovariectomy + high hard diet). It was a 2–month experiment. Enzyme–linked immunosorbent assay (ELISA) detected serum estradiol (E2), osteocalcin (BGP) and alkaline phosphatase (ALP) in rats. Bone histomorphometric indices in the third molar region of maxilla were detected by micro-CT; protein expressions of OPG, RANKL, and MGF in the third molar region of maxilla was detected by Western blot; and gene expression of OPG, RANKL, and MGF in the third molar region of maxilla was detected by Quantitative Real–Time PCR.

**Results:**

Comparing with model group, serum E2 in experimental group increased but not significantly, serum BGP and serum ALP in experimental group decreased but not significantly, OPG in experimental group in alveolar bone increased significantly, RANKL in experimental group in alveolar bone decreased significantly, RANKL/OPG ratio in experimental group decreased significantly, MGF in experimental group in alveolar bone increased significantly, bone volume to total volume fraction increased significantly in experimental group, trabecular thickness increased significantly in experimental group, and trabecular separation decreased significantly in experimental group.

**Conclusion:**

Enhanced masticatory force affected the expression of OPG, RANKL, and MGF in alveolar bone of ovariectomized rats, improved the quality of jaw bone of ovariectomized rats, and delayed oral bone loss by ovariectomy.

## Introduction

Menopause results in oral bone loss,^1^^–^^3^ which can lead to loose tooth, tooth loss, and periodontal disease.^2^^–^^4^ Kribbs^5^ (1990) found women with osteoporosis were three times more susceptible for teeth loss than their healthy controls. As reported, approximately 200 million women are affected by osteoporosis worldwide, which includes one third of 60 to 70–year–old women and two thirds of over 80 year–old women.^6^ Skeletal osteoporosis results in lower bone mineral density of women's mandibles.^7^ Oral bone loss induced by menopause is now a highly discussed topic among scholars.

Currently, three main methods are used to prevent and treat oral bone loss: drugs, surgery and physiotherapy.^8^^–^^13^ Many scholars investigated drugs for oral bone loss.^8^^–^^9^ A physical method of prevention and treatment, biomechanical therapy has drawn attention of scholars because of its noninvasive and non-side effects. Mavropoulos, et al.^11^^–^^13^ (2004, 2010, 2014) studied the effects of normal masticatory force on alveolar bone morphology of growing, normal adults and ovariectomized rats. Grünheid, et al.^14^ (2011) reported the effects of chewing force on jaw muscle and jaw mineralization in New Zealand white rabbits. Denes, et al.^15^ (2013) investigated the effect of reducing masticatory force on the alveolar bone and the molar periodontal ligament in the rat maxilla. The studies show chewing force affects oral and maxillofacial tissues and alters the microstructure of alveolar bone.

Many studies have shown OPG/RANK/RANKL is an important signal transduction pathway^16^^,^^17^, regulating bone remodeling, and plays an important role in regulating bone metabolism. OPG is expressed in many tissues and organs including osteoblasts, heart, bone marrow etc.; RANKL is also expressed in several tissues and organs such as osteoblast, skeletal muscle etc. MGF is an important mechanical signal transduction factor, expressed in response to mechanical stimulation and is important to maintain bone mass.^18^^,^^19^ MGF is found in many tissues, including osteoblast, skeletal muscle etc.

Masticatory may be a good biomechanical therapy for oral bone loss. In this study, menopausal rat model was established by ovariectomy, and enhanced masticatory force was simulated by feeding with a high hard feed. The effect of increased chewing force on the expression of OPG, RANKL, and MGF in alveolar bone was analyzed. Mechanical signal transduction in alveolar bone mechanical response to increased mastication forces was revealed at the molecular genetic level, and the effect of enhanced masticatory force on jaw. This study will help in enhancing mastication force to resist the loss of jawbone caused by menopause.

## Methodology

### Experimental animals

Laboratory Animal Center of Dalian Medical University, China [Quality certificate number: SCXK (Liao) 2008–0002] provided us with healthy specific pathogem free (SPF) female Sprague Dawley (SD) rats, with well-aligned dentition, 2–month–old, weighing 200–240 g. Two kinds of hard feed, normal hard feed (hardness 123 N, cylinder, 12 mm diameter, 20-40 mm length, water 9.6%) and high hard feed (hardness 216 N, cylinder, 12 mm diameter, 20-40 mm length, water 9.6%) with the same nutritional composition (according to Chinese Standard GB14924.3-2010) were customized in Jiangsu Xietong Pharmaceutical Bioengineering Co., Ltd. The study was approved by the ethics committee of Affiliated Zhongshan Hospital of Dalian University. The treatment of animals during the experiment followed the regulations of animal ethics standards of “Regulation for the Administration of Affairs Concerning Experimental Animals” issued by State Science and Technology Commission of China.

### Reagents and instruments

The following reagents and instruments were used: PVDF (polyvinylidene fluoride) membrane (Millipore, Temecula, California, USA); antibodies against OPG, RANKL, MGF and β-actin (Abcam, Cambridge, UK); horseradish peroxidase–conjugated anti-rabbit IgG anti-mouse IgG (Santa Cruz Biotechnology, Santa Cruz, California, USA); cell incubator, super clean bench (Forma Scientific Company, Marietta, Ohio, USA); cryogenic centrifuge (Sigma, Darmstadt, Germany); gel image analyzer (UVP, Upland, California, USA); automatic camera inversion microscope (Nikon, Tokyo, Japan), electronic balance (Sartorius, Germany), microdosimeter (Gilson, France), deionized water meter (Pall, Ann Arbor, MI, USA), microscopic CT machine (Siemens, Erlangen, Germany), centrifuge (Eppendorf, Hamburg, Germany), biospectrophotometer (Eppendorf, Hamburg, Germany), fluorescence–based quantitative PCR (Shanghai Hongshi, Shanghai, China).

### Experimental methods

#### Grouping and processing

Thirty SD rats were randomly divided into three groups: sham–operation group, model group, and experimental group. Before the experiments, all rats were acclimatized for one week. For the sham–operation group, only little fat around the ovary was removed without ovariectomy, while the other rats were bilaterally ovariectomized using a dorsal approach. One week later, the ovariectomized rats were divided into model group and experimental group. Rats in sham–operation group and model group continued to be fed with normal hard diet, while rats in experimental group were fed with high hard diet. All rats had free access to food and water. It was a 2–month investigation.

#### Tissue preparation

All rats were killed two months after fed by grouping. Before this, all rats stopped to eat and kept drinking water for 12–24 hours.

Serum samples: Blood samples were taken from the abdominal aorta, after a 30–minute period of rest at room temperature, centrifuged at 3,000 rpm for 15 minutes. Then, the supernatant was harvested and stored at −80°C for reserve.

Maxilla samples: Bilateral maxilla samples were extracted. After removing the soft tissue, one side of the maxillae was fixed in 10% neutral buffered formalin solution, and the other side was stored in refrigerator at −80°C for reserve.

Serum biochemical indicators

Serum E2 (estradiol), serum BGP (osteocalcin), and serum ALP (alkaline phosphatase) were determined by ELISA (Enzyme–linked immunosorbent assay) kit.

#### Bone histomorphometry

Maxillae of rats were evaluated using Siemens Inveon Micro-CT Scanner. Scanning conditions were as follows: voltage 80 kV, current 450 μA, resolution 15.48 um, and bone volume to total volume fraction (BV/TV) was measured, as well as trabecular number (Tb.N), trabecular separation (Tb.Sp), and trabecular thickness (Tb.Th).

#### OPG, RANKL and MGF proteins

Western blot was used to detect OPG, RANKL and MGF proteins expression in alveolar bone of the third molar region of the maxilla. Proteins were extracted by crushing and splitting bone tissue, separated by electrophoresis in 10% SDS-PAGE and then transferred to PVDF membrane. The blots were blocked with 5% skim milk and then incubated overnight at 4°C with primary antibodies. After washing, blotted membranes were incubated with the secondary antibody. Immunoreactive bands were developed by ECL method. Exposure films were scanned using gel image analyzer and quantified by gray value analysis with Image-Pro Plus software to measure the absorbance values of the target protein and the internal control β-actin protein. The relative expression level of target protein was normalized to the gray value of target protein/β-actin as an internal control. The mean value of three repeated tests was taken as the expression level of the target protein in the samples.

### OPG, RANKL and MGF mRNA

The gene expression of OPG, RANKL and MGF in alveolar bone of the third molar region of the maxilla were examined by quantitative real–time PCR. Maxillary third molars were extracted, using Trizol reagent to isolate total RNA. Integrity of RNA was analyzed using agarose gel electrophoresis, reverse transcription was performed with TIANScript RT Kit, and then quantitative amplification was performed using SuperRealPreMix Plus. Cyclic fluorescence signals of each reaction were monitored in real time, and cycle threshold (Ct) values were obtained. According to the ΔCt of target gene and housekeeping gene β-actin, model group was taken as control group, and statistical analysis was carried out by the relative quantitative method of 2^(−ΔΔCt). Each experiment was repeated three times.

Primer sequences: OPG gene, upstream CTGCACCTACCTAAAA, downstream GCAGCATTCAT, fragment size of 113 bp; RANKL gene, upstream AAAATCCCAAGTTCGCA, downstream GGACCTGGACGCTA, fragment size of 141 bp; MGF gene, upstream CCTCGTCAACTCAG, downstream TAGCAACATCCATCC, fragment size of 136 bp; β-actin gene, upstream CCTAGACTTCGAGCAAGAGA, downstream GGAAGGAAGCTGGAAGA, fragment size of 140 bp.

### Statistical analysis

SPSS 18.0 software was used for statistical analysis. All data were expressed by mean±standard deviation. Shapiro-Wilk test was used to examine for normal distribution and one-way ANOVA and LSD's *post-hoc* test, to evaluate data. Statistical significance was defined as p<0.05.

## Results

### Serum biochemical markers

**Figure 1:**
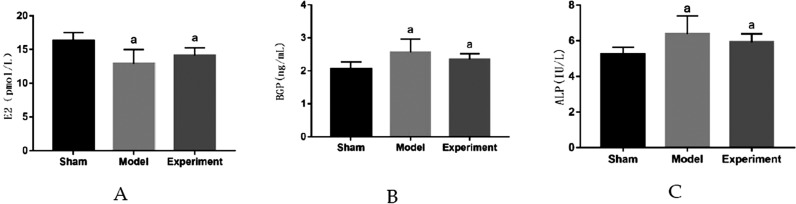
Test results of serum biochemical indicators in rats. ^a^p<0.05 significant differences in comparison with sham–operation group. A: E2; B: BGP; C: ALP

The distribution of serum biochemical indices in rats were normal, according to the Shapiro-Wilk test, for the sham–operated group (p=0.65 for serum E2, p=0.62 for serum BGP, p=0.70 for serum ALP), the model group (p=0.61 for serum E2, p=0.60 for serum BGP, p=0.69 for serum ALP) and experimental group (p=0.63 for serum E2, p=0.71 for serum BGP, p=0.66 for serum ALP). Figure 1 shows the result of serum biochemical indices in rats. Compared with sham–operated group, serum E2 in model group and experimental group decreased significantly (p<0.05), indicating that ovariectomy was successful; serum BGP and serum ALP in model group and experimental group increased significantly (p<0.05). Compared with model group, serum E2 in experimental group increased, serum BGP and serum ALP in experimental group decreased, but not significantly.

### Histomorphometric indices of bone

**Figure 2:**
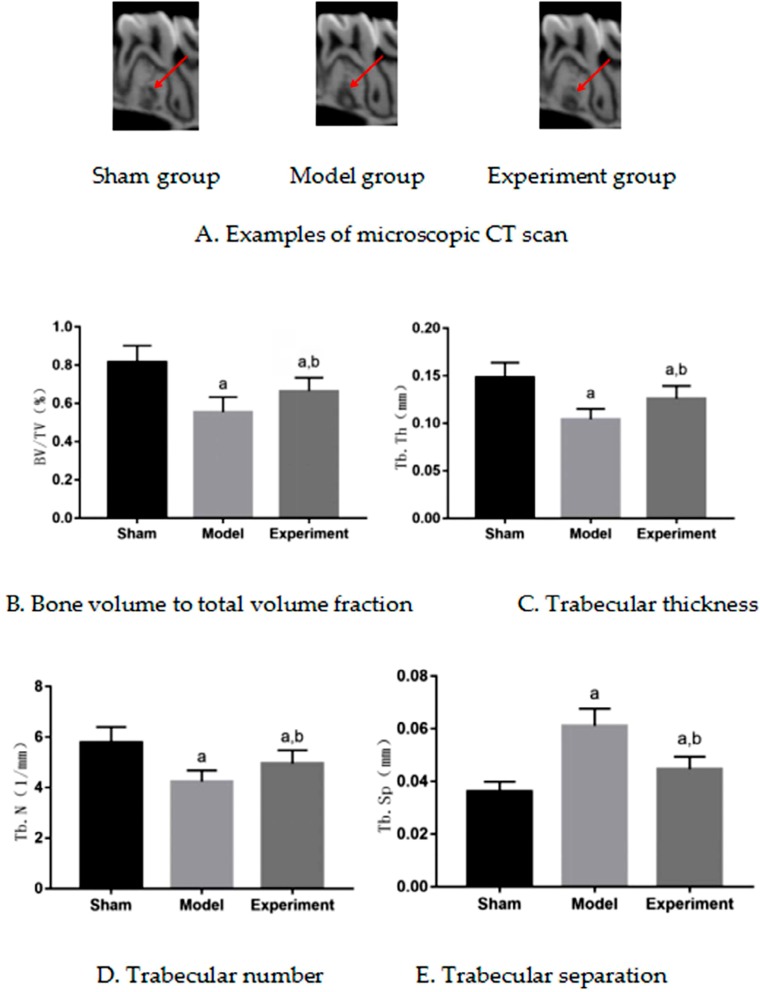
Microscopic cycle threshold (CT) results of rat maxilla. ^a^p<0.05 significant differences in comparison with sham group, ^b^p<0.05 significant differences in comparison with model group. A: microscopic CT scan (sham group, model group, experiment group); B: Bone volume to total volume fraction; C: Trabecular thickness; D: Trabecular number; E: Trabecular separation

The distribution of histomorphometric indices of bone in rats were normal, according to the Shapiro-Wilk test, for sham–operated group (p=0.59 for BV/TV, p=0.71 for Tb.Th, p=0.63 for Tb.N, p=0.66 for Tb.Sp), model group (p=0.68 for BV/TV, p=0.60 for Tb.Th, p=0.72 for Tb.N, p=0.64 for Tb.Sp) and experimental group (p=0.71 for BV/TV, p=0.68 for Tb.Th, p=0.59 for Tb.N, p=0.61 for Tb.Sp). Figure 2A shows micro-CT images of the maxilla M3. Compared with the sham–operated group, BV/TV, Tb.Th and Tb.N of model group decreased, and the Tb.Sp of model group increased (p<0.05); compared with model group, BV/TV, Tb.Th and Tb.N of experimental group increased, and Tb.Sp of experimental group decreased (p<0.05). Comparing with sham–operated group, experimental group had a significant difference (p<0.05). BV/TV, Tb.Th and Tb.N decreased, and Tb.Sp increased. Images of Figure 2A were taken at the same magnification and cut with Photoshop software by setting the height and width, being difficult to control the scanning cross section.

### OPG, RANKL, and MGF proteins

**Figure 3:**
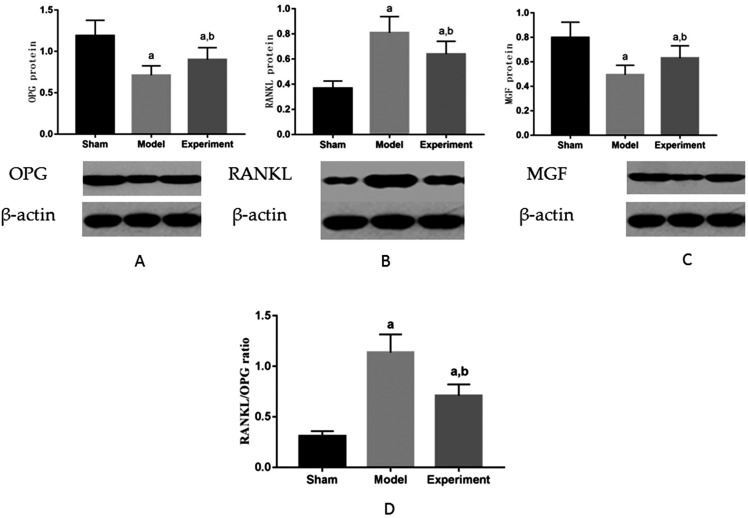
Expression of OPG, RANKL and MGF proteins in rat maxilla. ^a^p<0.05 significant differences in comparison with sham group, ^b^p<0.05 significant differences in comparison with model group. A: OPG; B: RANKL; C: MGF. D: RANKL/OPG ratio

The distribution of expression of OPG, RANKL, and MFG proteins in the maxilla M3 were normal, according to the Shapiro-Wilk test, for sham–operated group (p=0.56 for OPG, p=0.72 for RANKL, p=0.59 for MGF), model group (p=0.62 for OPG, p=0.54 for RANKL, p=0.63 for MGF) and experimental group (p=0.70 for OPG, p=0.65 for RANKL, p=0.57 for MGF). Figure 3 shows expression of OPG, RANKL, and MFG proteins in the maxilla M3. Compared with sham–operated group, OPG decreased, RANKL increased, RANKL/OPG ratio increased and MGF decreased in model group (p<0.05); compared with model group, OPG increased, RANKL decreased, RANKL/OPG ratio decreased and MGF increased in experimental group (p<0.05).

### OPG, RANKL and MGF gene

The distribution of expression of OPG, RANKL, and MFG gene in the maxilla M3 were normal, according to the Shapiro-Wilk test, for sham–operated group (p=0.73 for OPG, p=0.67 for RANKL, p=0.69 for MGF), model group (p=0.74 for OPG, p=0.63 for RANKL, p=0.66 for MGF) and experimental group (p=0.67 for OPG, p=0.61 for RANKL, p=0.58 for MGF).

**Figure 4:**
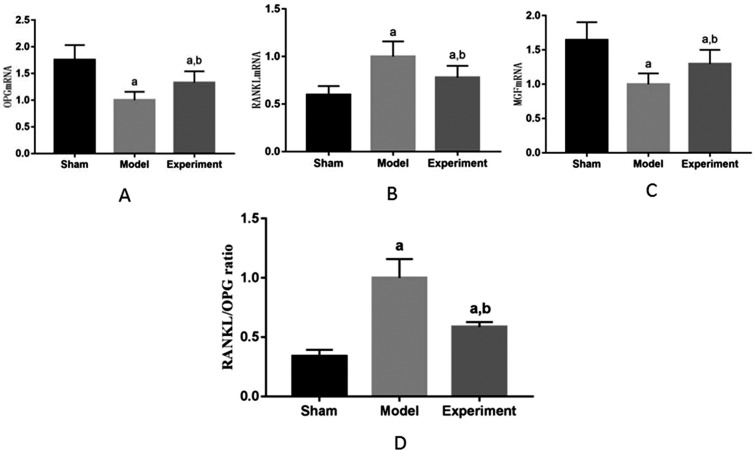
Detection of OPG, RANKL and MGF genes in rat maxilla. ^a^p<0.05 significant differences in comparison with sham group, ^b^p<0.05 significant differences in comparison with model group. A: OPG; B: RANKL; C: MGF; D: RANKL/OPG ratio

Figure 4 shows expressions of OPG, RANKL, and MFG gene in the maxilla M3. Compared with sham–operated group, OPG and MGF decreased, RANKL increased and RANKL/OPG ratio increased in model group (p<0.05), and the difference was statistically significant. Compared with model group, OPG and MGF increased, RANKL decreased and RANKL/OPG ratio decreased in experimental group (p<0.05), and the difference was statistically significant, which was consistent with the trend of protein expression.

## Discussion

### Effect of enhanced masticatory force on serum biochemical parameters in ovariectomized rats

Estrogen is the key hormone to maintain bone mass,^20^ and its reduction is the main cause of osteoporosis in postmenopausal women.^21^ Estrogen reduction results in microstructural changes in alveolar bone, and alveolar bone resorption is a local manifestation of osteoporosis in the oral field.^22^ Animal models for osteoporosis are usually established by removing ovaries to lower estrogen levels.^2^^,^^20^^–^^22^ Osteoporosis is related to oral bone loss in animal studies on rats and it results in lower bone mineral density of women's mandibles.^1^^–^^2^^,^^7^ In this study, when comparing with sham–operated group, serum E2 level in model group and experimental group decreased significantly, indicating ovariectomy was successful. Compared with model group, serum E2 level in experimental group increased, but not significantly. Many studies have shown that moderate exercise increased E2 levels.^23^^,^^24^ In this work, the results tended to be the same, and the difference in results was not significant.

BGP and ALP, which are mainly synthesized by osteoblasts, reflecting the activity of osteoblasts and the process of osteogenesis, are specific indicators of bone turnover.^25^^,^^26^ In this context, compared with sham–operated group, levels of BGP and ALP in model group and experimental group increased significantly, indicating that the bone remodeling was active and bone turnover rate was high after ovariectomy; compared with model group, levels of BGP and ALP in experimental group decreased, but not significantly, indicating that enhanced masticatory force inhibited bone turnover to a certain extent. Bone resorption was inhibited, and bone formation was enhanced, although not reaching the normal level. The reason for this difference may be that mastication movement is an oral facial movement involving teeth and jawbone, and muscles including the tongue and jaw muscles. Although it promotes myokines release, it does not eliminate the effect of estrogen reduction on bone. This suggests enhanced masticatory force might improve osteoporosis by adjusting E2, BGP and ALP levels to regulate the balance of bone remodeling.

### Effect of enhanced masticatory force on microstructure of maxillary bone in ovariectomized rats

Mechanical stimulation is the key factor to maintain or increase bone mass and strength.^27^ Mechanical stimulation can promote bone growth. Jawbone is the main masticatory organ, and it is the most metabolically active part of the skeletal system. The load on jawbone is mainly chewing force, and mastication promotes jaw growth and development. The evolutionary process of human beings has indicated that chewing force has a great influence on jaws evolution and development.^28^ Studies have shown that changes of masticatory force affect microstructure of alveolar bone in rats. High masticatory force improves alveolar bone microstructure, while low masticatory force leads to bone loss.^11^^,^^12^^,^^14^^,^^15^ After menopause, estrogen decreases, bone resorption increases, bone mass decreases, bone trabecula degenerates, and osteoporosis occurs.^29^ This study showed that BV/TV, Tb.Th and Tb.N of alveolar bone decreased significantly, and Tb.Sp of alveolar bone increased significantly after ovariectomy, which indicated that rats were in the process of osteoporosis. In this context, compared with model group, BV/TV, Tb.Th and Tb.N of alveolar bone in experimental group increased significantly, and Tb.Sp of alveolar bone in experimental group decreased significantly. This indicated that enhanced masticatory force appropriately increased bone mass and improved microstructure of alveolar bone.

### Effect of enhanced chewing force on MGF in maxillary bone in ovariectomized rats

Mechanical stimulation modulates gene expression and protein synthesis to regulate cell growth and differentiation.^30^ Some studies have found that MGF is a stress–sensitive factor, which is produced by alternative splicing of insulin–like growth factor-1 (IGF-1) under mechanical stress, and expression level of MGF is closely related to mechanical stimulation. Studies have shown expression of MGF is highly correlated with mechanical strain.^31^ This study showed that, compared with sham–operated group, MGF decreased significantly in model group and experimental group; compared with model group, MGF in experimental group increased significantly. Studies^32^^,^^33^ have shown level of IGF-1 in postmenopausal patients with osteoporosis decreased significantly, and IGF-1 was positively correlated with BMD and E2. MGF is a splice variant of IGF-1. E2 affects level of IGF-1, which may also affect expression level of MGF. In this work, MGF in experimental group increased significantly compared with model group, and then this showed enhanced chewing force promoted expression of MGF.

### Effect of enhanced chewing force on OPG/RANKL/RANK cytokine system in maxillary bone in ovariectomized rats

OPG/RANKL/RANK system plays an important role in regulating bone remodeling.^34^ The binding of RANK to RANKL induces the survival, proliferation, differentiation, and activation of osteoclasts.^35^^,^^36^ OPG also binds to RANKL by competing with RANK, and then protects the skeleton from excessive bone resorption. The expression levels of OPG and RANKL in local microenvironment of bone tissue are the key to maintain the balance between bone resorption and formation. Many studies have confirmed that mechanical stimulation regulates the expression of OPG and RANKL. The strength, frequency, and duration of mechanical stimulation are important factors affecting the expressions of OPG and RANKL. Mechanical stimulation increases the expression of OPG, inhibits the expression of RANKL, which reduces RANKL/OPG ratio, and then promote osteogenesis.^37^^,^^38^ In this study, compared with sham–operated group, OPG level in model group and experimental group decreased significantly, RANKL level in model group and experimental group increased significantly; compared with model group, OPG level in experimental group increased significantly, and RANKL level decreased significantly. Studies have shown estrogen regulated bone remodeling by regulating the OPG/RANK/RANKL signal axis.^39^ Findings of this study may prove this perspective. The results showed masticatory force affected the expression of OPG/RANKL and regulated the OPG/RANK/RANKL signaling pathway.

Xin^40^(2014) reported that MGF had a remarkable ability to regulate Wnt/β-catenin signaling pathway. From the results of this study, the conclusion is that MGF had some connection with Wnt/β-catenin signaling pathway. A significant inverse relationship occurs between MGF and RANKL/OPG ratio.

## Conclusions

In conclusion, our study demonstrated several important points relating to alveolar bone loss in ovariectomized rats. Firstly, enhanced masticatory force delayed degeneration of alveolar bone caused by ovariectomy, improved quality of jawbone in ovariectomized rats, and postponed loss of jawbone caused by ovariectomy. Secondly, enhanced masticatory force regulated levels of OPG, RANKL, and MGF in alveolar bone of ovariectomized rats and then affected bone remodeling balance. Thirdly, enhanced masticatory force regulated serum biochemical parameters in ovariectomized rats.

Mechanisms of bone remodeling is a complex biological process regulated by a variety of molecular signaling systems.^41^ Biomechanical signals transduction *in vivo* is also complex. Further studies are required for biomechanical signals transduction of local mechanical stimulation of enhanced masticatory force and influence of enhanced masticatory force on systemic indicators related to bone remodeling.
